# Assessment of chronic post-surgical pain after knee replacement: Development of a core outcome set

**DOI:** 10.1002/ejp.582

**Published:** 2014-08-25

**Authors:** V Wylde, F MacKichan, J Bruce, R Gooberman-Hill

**Affiliations:** 1 Musculoskeletal Research Unit, School of Clinical Sciences, Southmead HospitalBristol, UK; 2 Warwick Clinical Trials Unit, University of WarwickCoventry, UK

## Abstract

**Background:**

Approximately 20% of patients experience chronic post-surgical pain (CPSP) after total knee replacement (TKR). There is scope to improve assessment of CPSP after TKR, and this study aimed to develop a core outcome set.

**Methods:**

Eighty patients and 43 clinicians were recruited into a three-round modified Delphi study. In Round 1, participants were presented with 56 pain features identified from a systematic review, structured interviews with patients and focus groups with clinicians. Participants assigned importance ratings, using a 1–9 scale, to individual pain features; those features rated as most important were retained in subsequent rounds. Consensus that a pain feature should be included in the core outcome set was defined as the feature having a rating of 7–9 by ≥70% of both panels (patients and clinicians) and 1–3 by ≤15% of both panels or rated as 7–9 by ≥90% of one panel.

**Results:**

Round 1 was completed by 71 patients and 39 clinicians, and Round 3 by 62 patients and 33 clinicians. The final consensus was that 33 pain features were important. These were grouped into an 8-item core outcome set comprising: pain intensity, pain interference with daily living, pain and physical functioning, temporal aspects of pain, pain description, emotional aspects of pain, use of pain medication, and improvement and satisfaction with pain relief.

**Conclusions:**

This core outcome set serves to guide assessment of CPSP after TKR. Consistency in assessment can promote standardized reporting and facilitate comparability between studies that address a common but understudied type of CPSP.

**What's already known about this topic?**

- Around 20% of patients experience chronic pain after knee replacement.
- There is no agreement in the research literature about which aspects of pain should be assessed in studies of chronic pain after knee replacement.

**What does this study add?**

- This study developed an 8-item core outcome set to inform the assessment of chronic pain after total knee replacement.

## 1. Introduction

Primary total knee replacement (TKR) is one of the most common surgical procedures, and the future need for the operation has been predicted to increase (Kurtz et al., 2007). In 2012, over 75,000 TKRs were performed by the UK National Health Service (National Joint Registry, 2013). In the United States, 4,000,000 people aged over 50 currently live with a TKR (Weinstein et al., 2013). The operation is primarily performed to provide relief from chronic pain and improve function (National Joint Registry, 2013). Although a successful operation for many patients, approximately 20% of patients experience chronic post-surgical pain (CPSP) after knee replacement (Beswick et al., 2012). CPSP is defined as pain persisting for 3 months or more after surgery (Werner and Kongsgaard, 2014).

Accurate assessment of this condition is important given its prevalence and the distress that it can cause (Jeffery et al., 2011). The Initiative on Methods, Measurement, and Pain Assessment in Clinical Trials (IMMPACT) provides a framework to guide outcome assessment in trials of chronic pain treatments (Turk et al., 2003), recommending that assessment of pain should include intensity, temporal components, use of rescue treatments and quality (Dworkin et al., 2005). Despite the existence of this framework, a systematic review identified ongoing inconsistency and variability in the assessment of CPSP after TKR, with few studies adopting a comprehensive approach (Wylde et al., 2013). Reasons for nonadherence to evidence-based recommendations are complex and multifactorial with contributing factors including a lack of awareness and the perception that recommendations may not apply in certain settings (Lugtenberg et al., 2009). This suggests that condition-specific, evidence-based recommendations may be beneficial in promoting more robust assessments of pain and reducing variation in outcomes assessment.

There has been increasing interest in the development of core outcome sets to improve outcomes assessment, as demonstrated by the Core Outcome Measures in Effectiveness Trials (COMET) initiative (Williamson and Clarke, 2012). A core outcome set is an agreed standardized set of outcomes that should be measured and reported, as a minimum, in all research studies on a particular treatment or condition (Sinha et al., 2011). Core sets are developed to guide selection of appropriate outcome measures and ensure that outcomes important to key stakeholders are assessed (Gandhi et al., 2008). Standardization of outcomes assessment can reduce heterogeneity between trials, thereby aiding data synthesis (Clarke, 2007) and minimizing the risk of outcome reporting bias (Williamson et al., 2005). The Delphi method is commonly used to achieve consensus for a core outcome set (Jones and Hunter, 1995; Sinha et al., 2011). This is a structured technique in which participants with relevant expertise complete sequential rounds of questionnaires to reach consensus. This method has been used to develop core outcome sets in many clinical settings, including eczema (Schmitt et al., 2011), maternity care (Devane et al., 2007), childhood asthma (Sinha et al., 2012), systemic sclerosis (Khanna et al., 2008) and fibromyalgia (Mease et al., 2008). The aim of this study was to develop a core outcome set for the assessment of CPSP after TKR using a modified Delphi method.

## 2. Participants and methods

Ethics approval was provided by the South West – Central Bristol National Research Ethics Committee in England, UK (13/SW/0045), and all participants gave informed written consent. The study was registered on the UKCRN (United Kingdom Clinical Research Network) portfolio (ID 13903) and the COMET website database.

A patient panel and a clinician panel were established to ensure that differing views were represented in the final consensus (Sinha et al., 2011). Patients and clinicians were included at a ratio of 2:1 to reflect the importance of patients' views in the development of this pain-based core outcome set (Williamson et al., 2012). A single heterogeneous panel of clinicians, rather than multiple homogeneous panels grouped by discipline, was established to reflect the integrated and multidisciplinary approach required for the treatment and management of CPSP after TKR.

### 2.1 Patient panel

Patients who experienced CPSP in their replaced knee were eligible to participate in the study. Patients experiencing CPSP at a minimum of 1 year post-operatively were identified from other orthopaedics studies within the Research Unit (Wylde et al., 2011, 2012; Howells et al., 2014) through reviewing patient self-complete study questionnaires. Patients were eligible if they reported experiencing moderate, severe or extreme pain on to at least one item on the 5-item WOMAC (Western Ontario and McMaster University Arthritis Index) pain scale (Bellamy et al., 1988). Eligible patients were sent a study pack and patients interested in participating were asked to complete and return a consent form, reply slip and screening questionnaire. The screening questionnaire included questions about demographics, socioeconomic status and two validated patient-reported outcome measures to assess current knee-related pain and disability: the Oxford Knee Score (Dawson et al., 1998) and Chronic Pain Grade (Von Korff et al., 1992). The Oxford Knee Score is a 12-item joint-specific questionnaire designed to assess pain and functional limitations in patients undergoing TKR, with total scores ranging from 12 to 60 (best–worst). The Chronic Pain Grade is a 7-item questionnaire which assesses pain persistence, pain intensity and pain-related disability. Scoring results in patients being classified into one of five hierarchical pain grades, ranging from 0 (pain free) to IV (high disability–severely limiting).

### 2.2 Clinician panel

Clinicians in the United Kingdom and overseas with experience of caring for patients with CPSP after TKR were eligible to participate in the study. Identification of national and international participants for the clinician panel followed published recommended guidance regarding the procedure for selecting experts for a Delphi panel (Okoli and Pawlowski, 2004). Participants were recruited through four methods. First, relevant professional organizations were contacted and organizational gatekeepers were asked to disseminate study information via e-mail. Organizations included the British Pain Society, British Orthopaedic Association, British Association for Surgery of the Knee, Royal College of Nursing and the Association of Orthopaedic Chartered Physiotherapists. Second, clinicians who participated in a focus group study (Mackichan et al., 2014) which informed the selection of pain features for the Delphi study were approached about participation. These clinicians were based within a single National Health Service Trust and had experience of working with patients with CPSP after TKR. Third, a sample of clinicians identified in a nonsystematic search of the research literature as having previously published one or more articles on CPSP after TKR were sent information about the study. Fourth, a snowball technique was used whereby participants were asked to provide contact details for other clinicians with relevant experience who may be interested in participating, and these nominated clinicians were sent information about the study. Individuals interested in participating in the study were asked to complete and return a screening questionnaire, reply form and consent form. The screening questionnaire included questions about demographics, professional background and current job role.

### 2.3 Sample size

The sample size for a Delphi study depends on group dynamics for obtaining consensus among experts, rather than statistical power (Okoli and Pawlowski, 2004). There is no set guidance available as to how many participants should be included in a Delphi study (Sinha et al., 2011). A minimum of 10 participants on a panel has been suggested (Okoli and Pawlowski, 2004), and previous studies using the Delphi method to create a core outcomes set have included between 13 and 222 participants (Sinha et al., 2011). To ensure the incorporation of a range of views and opinions, 123 people were recruited into this study (80 patients and 43 clinicians).

### 2.4 Identification of pain features and development of the Delphi questionnaire

**Figure 1:**
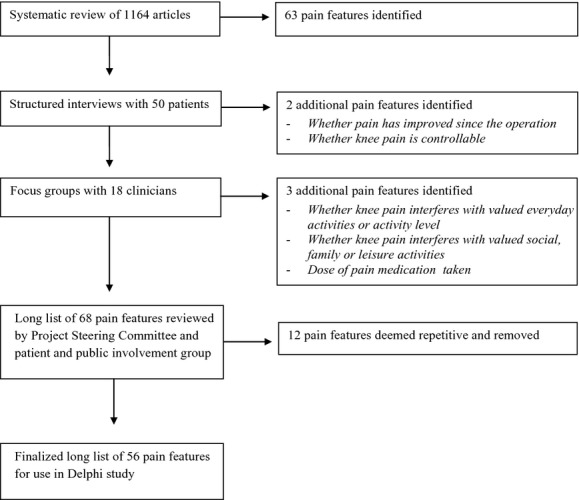
Development of a long list of pain features.

A modified Delphi method involves preliminary work to identify outcomes and then administrating a structured questionnaire to participants in consecutive rounds. Identification of pain features for inclusion in the Delphi study was through three sources: a systematic review of 1164 published articles which assessed CPSP after TKR (Wylde et al., 2013), structured interviews with 50 patients with CPSP after TKR (Howells et al., 2014) and four focus groups with 18 clinicians involved in providing care for joint replacement patients (Mackichan et al., 2014). Data about the assessment of pain were extracted from these sources and coded into pain features. Coding was performed in duplicate by two researchers and pain features were then refined and modified through further discussion and review. The resulting 68 pain features were further refined through discussion with a patient and public involvement group specializing in musculoskeletal research [Patient Experience Partnership in Research; PEP-R (Gooberman-Hill et al, 2013)] and the Project Steering Committee. The PEP-R group is facilitated by a patient involvement coordinator and involves 12 patients with musculoskeletal conditions, most of whom have had joint replacement. The Project Steering Committee consisted of an orthopaedic surgeon, pain clinician, two patient representatives and a patient involvement coordinator. Twelve pain features were deemed repetitive and therefore removed, resulting in the inclusion of 56 pain features in the Round 1 questionnaire. The pain features were written in plain English language and where appropriate, medical terminology was included in parentheses afterwards e.g., ‘Knee pain when bending the knee (pain on flexion)’. Modifications were also made to reduce and clarify the questionnaire instructions based on the feedback from the PEP-R group. A flow diagram depicting the process involved in the development of the long list of pain features, and the number of pain features identified from each source, is presented in Fig. 1.

The Delphi questionnaires were administered to members of the clinician panel by electronic mail to facilitate inclusion of international participants, and to members of the patient panel by post. The three rounds of questionnaires were administered over a 4-month period during 2013, with a period of approximately 5 weeks between each questionnaire. Participants of both panels were sent the same questionnaires, followed by a reminder if no response was received after 3 weeks. Nonresponders were not invited to participate in subsequent rounds.

### 2.5 Round 1

For each pain feature in the Delphi questionnaire, participants were asked ‘In your opinion, how important is it to ask patients with long-term pain after knee replacement about this pain feature/quality?’ Participants then rated the importance of assessing each pain feature on a numeric rating scale from 1 (anchored with the wording ‘not important’) to 9 (anchored with the wording ‘very important’).

#### 2.5.1 Round 1 analysis

Descriptive statistics were used to summarize the results of Round 1. Analysis was conducted separately for the patient panel and clinician panel to identify discrepancies. The criteria for retaining pain features between rounds and the definition of consensus were specified *a priori* in the study protocol. The same criteria were used for both and were based on published recommendations for defining consensus (Williamson et al., 2012). Median scores for each pain feature were calculated. Based on the GRADE guidelines (Guyatt et al., 2011), pain features with a median scores of 1–3 were considered as having limited importance, pain features with a median score of 4–6 as important but not critical and those with a score of 7–9 as critically important. Pain features given an importance rating of 7–9 by ≥70% of members of both panels and rated as 1–3 by ≤15% of members of both panels were retained and carried forward to Round 2. To ensure that pain features considered exceptionally important by only one panel were not omitted, we added the additional criterion that features rated as 7–9 by ≥90% of members of one panel, regardless of the ratings of the other panel, were also carried forward to Round 2. Participants were not informed of the specific criteria for retention of pain features between rounds or the definition of consensus, but they were informed that the most important pain features would be retained and included in the core outcome set.

### 2.6 Round 2

Participants were sent a Round 2 questionnaire which contained the pain features retained from Round 1. Within the questionnaire, participants were provided with the median ratings from the patient and clinician panels and their own Round 1 ratings. They were asked to again rate the importance of the listed pain features. Participants were free to change their ratings from Round 1 or keep them the same.

#### 2.6.1 Round 2 analysis

Pain features were retained and carried forward to Round 3 if they were given an importance rating of 7–9 by ≥70% of both panels and 1–3 by ≤15% of both panels, or rated as 7–9 by ≥90% of one panel.

### 2.7 Round 3

Participants were sent a Round 3 questionnaire which contained the pain features retained from Round 2. For each pain feature, participants were asked to rate if it should be included in a core outcome set, by giving it a rating between 1 (complete disagreement that the feature should be included in a outcome set) and 9 (complete agreement feature should be included in a core outcome set).

#### 2.7.1 Round 3 analysis

Consensus that a pain feature should be included in the core outcome set was defined as the feature having a rating of 7–9 by ≥70% of both panels and 1–3 by ≤15% of both panels, or rated as 7–9 by ≥90% of one panel.

### 2.8 Development of the core outcome set

The pain features that were retained after the three rounds of the Delphi study were reviewed and systematically categorized into core outcome domains by members of the research team (Macefield et al., 2014). The IMMPACT recommendations (Dworkin et al., 2005) were used as a broad framework for this process. Each individual feature was reviewed to determine whether it was appropriate to group it into an IMMPACT recommended pain outcome (pain intensity, the use of rescue treatments, pain quality, temporal components of pain) or a new pain outcome domain. The developed outcomes domains were then reviewed to ensure that the features they encompassed adequately reflected the domain and that the features were conceptually similar. These core outcome domains were subsequently discussed and refined by the Project Steering Committee and the PEP-R group.

## 3. Results

### 3.1 Patient panel

Study information packs were sent to 223 patients and 80 consented to participate, giving a recruitment rate of 36%. Nonparticipants had a mean age of 72 years (95% confidence intervals: 70–73) and 62% were female, which was similar to the demographics of participants (Table 1). Only participants who completed the previous round were invited to participate in subsequent rounds; 71 of 80 patients (89%) completed Round 1, 67 of 71 (94%) completed Round 2 and 62 of 67 (93%) completed Round 3. On average, patients who dropped out between rounds gave lower importance ratings than those who completed the round (Supporting Information Table S1). Characteristics of patients and participants who completed all three rounds of the Delphi study are provided in Table 1.

**Table 1:** Characteristics of members of the patient and clinician panels

	Recruited	Completed Delphi study
**Patient panel**		
Number	80	62
Mean age in years (range)	73 (55–91)	72 (56–85)
Gender (% female)	56	57
Mean years post-operative (range)	5 (1–10)	5 (1–10)
Ethnicity (% white)	99	100
Living status (% living with other(s))	78	79
Education (% with college or university education)	27	23
Work status (% retired)	85	84
Mean Oxford knee score (range)	36 (21–56)	36 (21–56)
Chronic pain grade (%)		
Grade 0: Pain free	0	0
Grade I: Low disability-low pain intensity	30	30
Grade II: Low disability-high pain intensity	16	18
Grade III: High disability-moderately limiting	32	33
Grade IV: High disability-severely limiting	22	20
**Clinician panel**		
Number	43	33
Mean age in years (range)	43 (29–58)	42 (29–58)
Number of females (%)	22 (51%)	18 (55%)
Country of work (number)		
UK	39	29
Australia	2	2
Canada	2	2
Mean years experience in profession (range)	19 (3–35)	19 (3–35)
Profession (number)		
Allied health professional	26	21
Orthopaedic surgeon/rheumatologist	11	9
Pain clinician	6	3

### 3.2 Clinician panel

Forty-three clinicians with experience of caring for patients with CPSP after TKR consented to participate in the study. Of these clinicians, 15 were recruited through professional organizations (recruitment was through advertising and therefore the recruitment rate is unknown), 15 through personalized invitations to clinicians identified from the research literature (33 approached, recruitment rate of 45%), eight from a previous focus group study (18 approached, recruitment rate of 44%) and five through snowball sampling (15 approached, recruitment rate of 33%). Participants consisted of 23 physiotherapists, 10 orthopaedic surgeons, six pain clinicians, two nurses, one rheumatologist and one occupational therapist. Of the recruited clinicians, 39 of 43 (91%) completed Round 1, 35 of 39 (90%) completed Round 2 and 33 of 35 (94%) completed Round 3. On average, clinicians who dropped out between rounds gave lower importance ratings than those who completed the round (Supporting Information Table S1). Characteristics of recruited clinicians and participants who completed all three rounds of the Delphi study are provided in Table 1.

### 3.3 Round 1

In Round 1, participants gave importance ratings to the 56 pain features identified through the systematic review of the literature, structured interviews with patients and focus groups with clinicians. The highest and lowest rated pain features in each round, ranked by panel patient ratings, is provided in Supporting Information Tables S2–S4. On average, the patient panel assigned higher importance ratings to pain features than the clinician panel. In Round 1, all 56 pain features were given a median rating of 7–9 by the patient panel, compared with 44 pain features by the clinician panel. There was consensus between the patient and clinician panel that 32 features should be retained and carried forward to Round 2. In addition, although the feature ‘pain when kneeling’ was rated as important by only 21% of clinicians, ≥ 90% of patients rated this feature as important and therefore it was carried forward to Round 2. A total of 33 pain features were retained and carried forward to Round 2. A full breakdown of the Round 1 results can be found in Supporting Information Table S2.

### 3.4 Round 2

In Round 2, participants again rated the importance of the 33 pain features after being provided with median panel ratings and their own personal ratings from Round 1. There was little change in the ratings of the pain features from Round 1 to Round 2, with consensus that 33 pain features should be retained and carried forward to Round 3. The pain feature ‘pain when kneeling’ was again carried forward to Round 3 because it was rated as important by ≥90% of the patient panel. A full breakdown of the Round 2 results can be found in Supporting Information Table S3.

### 3.5 Round 3

In this round, participants rated whether each of the 33 pain features carried forward from Round 2 should be included in the core outcome set. There was consensus that all 33 pain features should be included in the core outcome set. Three pain features were included in the core outcome set because they were rated as important by ≥90% of the patient panel; these included pain when kneeling, whether the pain is unbearable, and pain quality. A full breakdown of the Round 3 results can be found in Supporting Information Table S4.

### 3.6 Development of the core outcome set

The 33 pain features were reviewed by the research team and preliminarily categorized into core outcome domains. These groupings were discussed and refined during two meetings: one with the Project Steering Committee and one with the PEP-R group. Refinements included changing the names of a number of the core outcomes and recategorizing some pain features into more appropriate core outcomes. Details of these refinements are provided in Supporting Information Table S5. Final consensus that the 33 pain features should be grouped into an 8-item core outcome set was achieved (Table 2). The finalized core outcome set for CPSP after TKR consisted of the following pain domains: pain intensity, pain interference with daily living, pain and physical functioning, temporal aspects of pain, pain description, emotional aspects of pain, use of pain medication, and improvement and satisfaction with pain relief.

**Table 2:** Eight-item core outcome set for chronic post-surgical pain after total knee replacement

Core outcome	Pain features within core outcome^a^
Pain intensity	Average pain intensity
	Worst pain intensity
	Whether pain is controllable
Pain interference with daily living	Whether pain interferes with work or housework
	Whether pain interferes with valued everyday activities
	Whether pain interferes with valued social, family or leisure activities
	Whether pain interferes with walking
	Whether pain interferes with quality of life
	Whether pain interferes with rest or sleep
Pain and physical functioning	Pain with general activity
	Pain when walking
	Pain when kneeling
	Pain when climbing stairs
	Pain when descending stairs
	Whether pain is disabling
Temporal aspects of pain (time and pain)	Frequency of pain
	Duration of pain since the operation
	Night pain
	Constant pain
Pain description	Pain location
	Pain quality (e.g., sharp, aching, throbbing)
Emotional aspects of pain	Whether pain is unbearable
	Pain self-efficacy
	Ability to cope with pain
	Kinesiophobia
Use of pain medications	Frequency of pain medication use
	Reduced need for pain medications
	Type of pain medication taken
	Dose of pain medication taken
Improvement and satisfaction with pain relief	Whether expectations of pain relief have been met
	How pain compares to preoperative pain
	Whether pain has improved since the operation
	Satisfaction with pain relief from the operation

a The pain features are included in this table to demonstrate how the 8-item core outcome set was developed from the 33 pain features.

## 4. Discussion

IMMPACT recognizes the multidimensional nature of pain and recommends that six core outcomes should be considered in the design of all clinical trials investigating treatments for chronic pain (Turk et al., 2003). These recommended outcomes, developed during a consensus meeting involving 27 professionals, include pain, physical functioning, emotional functioning, participant rating of global improvement, symptoms and adverse events and participant disposition. IMMPACT provides further recommendations that the assessment of pain within clinical trials should include measures of pain intensity, the use of rescue treatments, pain quality and the temporal components of pain (Dworkin et al., 2005). Recent work has used the IMMPACT recommendations as a framework to identify key outcome features for epidemiological studies of all types of CPSP, recommending that assessment incorporates pain, physical functioning, psychological functioning and global ratings of outcome (VanDenKerkhof et al., 2013). Despite the publication of the IMMPACT recommendations in 2005, there remains a lack of comprehensive assessment of pain within the field of orthopaedic surgery. A systematic review of over 1000 studies reporting the outcome of TKR found major omissions in the assessment of CPSP after TKR (Wylde et al., 2013). Many studies failed to include any assessment of pain and when pain was measured, the assessment was oriented primarily towards pain severity with few studies using established pain assessment tools that would include assessment of pain impact or pain interference. There is some evidence in the orthopaedic literature of a move away from clinical outcomes of success, such as prosthetic survivorship, towards comprehensive assessment of pain and other patient-reported outcomes (Wylde and Blom, 2011). However, many studies still rely on clinician-administered tools, such as the American Knee Society Score (AKSS) (Insall et al., 1989), for outcome assessment (Riddle et al., 2008; Wylde et al., 2013). The AKSS is a composite score based on assessment of pain, functional ability and measurements such as range of motion and joint stability; pain is assessed by a single question focusing on severity. Given that most patients undergo a joint replacement in order to relieve chronic pain, it is surprising that pain – particularly the features that matter most to patients – is not at the centre of outcome assessment. This highlights the need for further work to improve the assessment of pain outcomes after TKR as well as for closer engagement between orthopaedic and pain research.

The 8-item, condition-specific core outcome set developed in this study supports current IMMPA CT recommendations on pain assessment, with the core outcome domains closely reflecting those recommended by IMMPACT (Dworkin et al., 2005). Our work provides evidence of the appropriateness of the generic IMMPACT recommendations in the field of CPSP. The IMMPACT core outcome domains were developed through consensus work among professionals only and our findings suggest that the domains also reflect patients' priorities. The development of this core outcome set also reinforces the view that pain assessment needs to be multidimensional and comprehensive. Pain severity, the primary outcome of interest in many studies of CPSP after TKR (Wylde et al., 2013), is only one of a multitude of features important to both patients and clinicians. Our work reiterates the importance of comprehensive assessment, given the complex processes involved in the aetiology, treatment and management of chronic pain (Dansie and Turk, 2013). The biopyschosocial model is widely accepted among pain clinicians, although this broader approach to understanding the multiple perspectives arising from persisting, distressing pain may not be the focus within orthopaedic surgery. Given that many orthopaedic surgical procedures are performed to treat chronic pain, there is scope to improve pain assessment.

It is important to acknowledge the limitations of this study when interpreting the results. Members of the patient panel were recruited from a single hospital and were mainly of white ethnicity. The lack of diversity in the sample population suggests that caution should be exercised when applying the core outcome set to other settings, given that pain perception and experience are influenced by ethnicity and cultural factors (Edwards et al., 2001). Similarly, the entire patient panel and the majority of the clinician panel were based in the United Kingdom and therefore this core outcome set was predominantly driven by consensus among people living in the United Kingdom. Further research would be required to determine if similar results would be obtained in the context of other settings. The use of a snowball technique of sampling to recruit clinicians had the potential for bias and we were unable to ascertain whether the members of the clinician panel were representative of those approached. The composition of the clinician panel also warrants consideration. The use of a single heterogeneous clinician panel meant that the views of physiotherapists, who comprised over half the panel, were strongly represented in the final consensus and the opinions of other health professionals with low representation on the panel, such as nurses and occupational therapists, would likely have been masked. In addition, although there was low attrition between rounds of the Delphi, participants who dropped out between rounds gave lower importance ratings compared with those who completed the round which may have introduced attrition bias and led to an overestimation of the level of consensus in the core outcome set (Sinha et al., 2011).

There is no ‘gold standard’ method for developing a core outcome set and therefore it is important to consider the advantages and disadvantages of the methods used. We used a modified Delphi method to develop the core outcome set. This involves conducting preliminary work to identify outcomes and then administration of a structured questionnaire to participants in Round 1. This differs from the traditional Delphi method which uses open-ended questions in the first round to elicit items from participants, with those items rated in subsequent rounds. Bias may have been introduced because participants were presented with pre-selected features (Sinha et al., 2011). However, the features were selected from numerous sources, including a systematic review and views of patients and experts. The modified Delphi method has several advantages over the traditional technique: in particular, the presentation of structured-closed questions is less burdensome to participants than open-ended questions (Hsu and Sandford, 2007). The use of strict criteria in Round 1 and 2 to determine which pain features were retained in the next round may have influenced the composition of the core outcome set; had less stringent criteria been used, more pain features may have been retained. In Round 2, participants were provided with the median importance rating given to each pain feature in the previous round. However, they were not provided with distribution of these scores and therefore did not have information around the variability in scores to aid their decision making in Round 2.

Strengths of the study include the diverse approaches taken to identify a comprehensive selection of pain features for inclusion in the Delphi study and inclusion of a separate patient and clinician panel. To our knowledge, this is the first study to identify and involve a large number of patients (*n* = 80) with CPSP to inform the development of a pain core outcome set. Participation of patients and clinicians on a 2:1 ratio ensured that, rather than being driven by experts, patients' voices and experiences were central to the development of this core outcome set. Closer engagement between patients, clinicians and researchers could be beneficial in the development of core outcome sets in other surgical and medical specialities. Analysing the results of the Delphi by panel allowed the identification of conflicting views which may not have been apparent had the panels been combined. Average ratings given by the patient panel were consistently higher than those of the clinician panel, with patients uniformly reporting certain pain features to be of ‘critical importance’. This reflects literature reporting that patients often give health outcomes higher importance ratings than clinicians (Hewlett, 2003; Wylde et al., 2006). A particularly striking difference between panels was the perceived importance of ‘pain on kneeling’, which was one of the most important pain features to patients but of least importance to clinicians. The importance of this feature to patients warrants its inclusion, and had we only explored clinicians' views, this feature would have been absent from the final core outcome set.

In conclusion, this study has highlighted the importance of a comprehensive and multidimensional approach to the assessment of CPSP after TKR. This is reflected in the 8-item core outcome set, which recommends that pain assessment should include the following pain domains: pain intensity, pain interference with daily living, pain and physical functioning, temporal aspects of pain (time and pain), pain description, emotional aspects of pain, use of pain medication, and improvement and satisfaction with pain relief. This condition-specific core outcome set reflects IMMPACT assessment recommendations for clinical trials investigating treatments for chronic pain. The findings from this study have the potential to facilitate a move towards improving the quality and consistency of pain assessment within orthopaedic surgery. Such a move will be challenging, requiring a shift from current convention. Continued dialogue and engagement with pain experts will be central to this transition.

## Author contributions

All authors were involved in the concept and design of the study. V.W. was responsible for data collection and analysis. All authors were involved in the interpretation of data. V.W. drafted the manuscript and all authors revised it critically for important intellectual content and gave final approval for the version to be published. V.W. takes responsibility for the integrity of the work.

## Supporting Information

**Table S1.** Comparison of median importance ratings for participants who did and did not complete subsequent rounds. **Table S2.** Delphi study Round 1 results (ordered by the percentage of the patient panel giving an importance rating of 7–9). **Table S3.** Delphi study Round 2 (ordered by the percentage of the patient panel giving an importance rating of 7–9). **Table S4.** Delphi study Round 3 (ordered by the percentage of the patient panel giving an importance rating of 7–9). **Table S5.** Refinements to 8-item core outcome set after review by the Project Steering Committee (SC) and patient and public involvement group (PEP-R).
