# Supplementary material for: Assessment of chronic post-surgical pain after knee replacement: Development of a core outcome set
Source: Eur J Pain. 2014 Aug 25;19(5):611–20. doi: 10.1002/ejp.582 (PMC4409075; doi:10.1002/ejp.582)
Supplement: Supplementary file 1 — Table S1. Comparison of median importance ratings for participants who did and did not complete subsequent rounds. Table S2. Delphi study Round 1 results (ordered by the percentage of the patient panel giving an importance rating of 7–9). Table S3. Delphi study Round 2 (ordered by the percentage of the patient panel giving an importance rating of 7–9). Table S4. Delphi study Round 3 (ordered by the percentage of the patient panel giving an importance rating of 7–9). Table S5. Refinements to 8-item core outcome set after review by the Project Steering Committee (SC) and patient and public involvement group (PEP-R). [file ejp0019-0611-sd1.docx]

**TableS1: Comparison of median importance ratings for participants who did and did not complete subsequent rounds**

|  | **Patients** | | **Clinicians** | |
| --- | --- | --- | --- | --- |
|  | **Completed Round 2 (n=67)** | **Did not complete Round 2 (n=4)** | **Completed Round 2 (n=35)** | **Did not complete Round 2 (n=4)** |
| Median Round 1 importance rating for 56 pain domains | 8 | 7.5 | 8 | 7.8 |
|  | **Completed Round 3 (n=62)** | **Did not complete Round 3 (n=5)** | **Completed Round 2 (n=33)** | **Did not complete Round 2 (n=2)** |
| Median Round 2 importance rating for 33 pain domains | 9 | 8 | 8 | 7 |

**TableS2: Delphi study Round 1 results (ordered by the percentage of the patient panel giving an importance rating of 7-9)**

|  | **Patient panel (n=71)** | | | | | **Clinician panel (n=39)** | | | | | **Retained in Round 2** |
| --- | --- | --- | --- | --- | --- | --- | --- | --- | --- | --- | --- |
|  | Median rating | IQR* | % 7-9 rating | % 1-3 rating | Number^#^ | Median rating | IQR* | % 7-9 rating | % 1-3 rating | Number^#^ |  |
| 1. Knee pain when kneeling | 9 | 8-9 | 91 | 0 | 65 | 5 | 3-6 | 21 | 31 | 39 | Yes |
| 2. Whether knee pain interferes with walking | 9 | 8-9 | 91 | 0 | 70 | 8 | 7-9 | 95 | 0 | 38 | Yes |
| 3. Whether knee pain interferes with quality of life | 9 | 8-9 | 91 | 0 | 70 | 8.5 | 8-9 | 89 | 0 | 38 | Yes |
| 4. Whether pain has improved since the operation | 9 | 8-9 | 90 | 1 | 70 | 9 | 8-9 | 97 | 0 | 38 | Yes |
| 5. Whether knee pain is disabling | 9 | 8-9 | 89 | 1 | 70 | 8 | 6-9 | 74 | 3 | 38 | Yes |
| 6. Whether knee pain is unbearable | 9 | 8-9 | 89 | 1 | 70 | 8 | 6-8 | 73 | 8 | 37 | Yes |
| 7. Whether knee pain interferes with valued everyday activities | 8 | 7-9 | 88 | 1 | 69 | 8 | 8-9 | 92 | 0 | 38 | Yes |
| 8. Satisfaction with pain relief from the operation | 9 | 8-9 | 87 | 1 | 70 | 8 | 7-9 | 79 | 3 | 38 | Yes |
| 9. Knee pain when descending stairs | 8 | 7-9 | 87 | 1 | 69 | 7 | 6-8 | 72 | 5 | 39 | Yes |
| 10. Knee pain when walking | 8 | 7-9 | 86 | 1 | 69 | 8 | 7-9 | 95 | 3 | 39 | Yes |
| 11. Location of knee pain (e.g. kneecap, back of knee) | 9 | 8-9 | 86 | 0 | 70 | 8 | 7-9 | 89 | 3 | 37 | Yes |
| 12. Emotional impact of pain e.g. sickening, tiring | 8 | 7-9 | 86 | 3 | 69 | 7 | 5-8 | 61 | 8 | 36 | No |
| 13. Whether knee pain is troubling, distressing or bothersome | 8.5 | 7-9 | 86 | 3 | 70 | 8 | 6-8 | 68 | 0 | 37 | No |
| 14. Ability to cope with pain | 8.5 | 7-9 | 86 | 1 | 70 | 8 | 7-8 | 86 | 3 | 37 | Yes |
| 15. Numbness in the knee | 8 | 7-9 | 85 | 3 | 68 | 6 | 4-7 | 45 | 21 | 38 | No |
| 16. Fear of moving knee because of the pain | 8 | 7-9 | 85 | 4 | 68 | 8 | 7-9 | 78 | 3 | 37 | Yes |
| 17. Worst knee pain intensity | 8 | 8-9 | 84 | 0 | 70 | 8 | 7-9 | 79 | 0 | 39 | Yes |
| 18. Whether knee pain interferes with rest or sleep | 9 | 8-9 | 84 | 0 | 68 | 9 | 8-9 | 97 | 0 | 38 | Yes |
| 19. How pain compares to pre-operative pain | 9 | 8-9 | 84 | 0 | 70 | 9 | 7-9 | 92 | 0 | 38 | Yes |
| 20. Ability to carry on despite the pain | 8 | 7-9 | 84 | 3 | 70 | 8 | 7-8 | 84 | 3 | 37 | Yes |
| 21. Type of pain medication taken | 8 | 7-9 | 84 | 1 | 70 | 8 | 7-9 | 82 | 0 | 38 | Yes |
| 22. Dose of pain medication taken | 8 | 7-9 | 84 | 3 | 70 | 8 | 7-9 | 76 | 8 | 38 | Yes |
| 23. Frustration or annoyance caused by knee pain | 8 | 7-9 | 83 | 4 | 71 | 7 | 6-8 | 54 | 11 | 37 | No |
| 24. Whether knee pain is controllable | 8 | 7-9 | 83 | 3 | 69 | 8 | 7-9 | 86 | 0 | 37 | Yes |
| 25. Frequency of knee pain | 8 | 7-9 | 83 | 0 | 69 | 8 | 7-9 | 82 | 0 | 38 | Yes |
| 26. Whether knee pain interferes with valued social, family or leisure activities | 8 | 7-9 | 82 | 2 | 67 | 8 | 7-9 | 82 | 0 | 38 | Yes |
| 27. Knee pain when getting in and out of a car | 8 | 7-9 | 82 | 1 | 67 | 6 | 5-8 | 31 | 15 | 39 | No |
| 28. Frequency of pain medication use | 9 | 7-9 | 81 | 3 | 70 | 8 | 7-9 | 92 | 0 | 39 | Yes |
| 29. Whether knee pain interferes with work or housework | 8 | 7-9 | 81 | 1 | 69 | 8 | 7-9 | 89 | 0 | 38 | Yes |
| 30. Reduced need for pain medications | 8 | 7-9 | 81 | 3 | 69 | 8 | 7-9 | 81 | 3 | 37 | Yes |
| 31. Knee pain with general activity | 8 | 7-9 | 81 | 3 | 67 | 7 | 6-8 | 74 | 10 | 39 | Yes |
| 32. Pain quality (how the pain feels e.g. sharp, aching, cramping) | 8 | 7-9 | 81 | 1 | 68 | 7 | 6-8 | 74 | 3 | 38 | Yes |
| 33. Knee pain when climbing stairs | 9 | 7-9 | 81 | 1 | 69 | 7 | 6-8 | 71 | 5 | 38 | Yes |
| 34. Knee pain when rising from a chair | 8 | 7-9 | 81 | 1 | 69 | 7 | 6-8 | 67 | 10 | 39 | No |
| 35. Worry or upset caused by knee pain | 8 | 7-9 | 81 | 3 | 69 | 7 | 6-8 | 59 | 5 | 37 | No |
| 36. Whether expectations of pain relief have been met | 8 | 7-9 | 80 | 0 | 70 | 8 | 7-9 | 84 | 0 | 38 | Yes |
| 37. How long the pain has been present since the operation | 8 | 7-9 | 80 | 1 | 71 | 8 | 7-9 | 79 | 3 | 39 | Yes |
| 38. Knee pain when standing | 8 | 7-9 | 80 | 3 | 70 | 8 | 6-8 | 66 | 8 | 38 | No |
| 39. Knee pain when bending the knee (flexion) | 8 | 7-9 | 80 | 4 | 69 | 7 | 6-8 | 62 | 3 | 37 | No |
| 40. Pain when knee makes a loud clunking noise (pain with patellar clunking) | 8 | 7-9 | 80 | 3 | 69 | 7 | 5-8 | 58 | 8 | 38 | No |
| 41. Constant knee pain (pain that is there all the time) | 9 | 7-9 | 79 | 3 | 70 | 8 | 7-9 | 82 | 3 | 39 | Yes |
| 42. Knee pain that occurs first thing in the morning | 8 | 7-9 | 77 | 1 | 69 | 6 | 5-8 | 41 | 8 | 39 | No |
| 43. Knee pain when twisting/pivoting the knee | 8 | 7-9 | 77 | 1 | 69 | 5 | 5-7 | 32 | 11 | 37 | No |
| 44. Whether the pain is caused by problems with the nerve endings (neuropathic pain) | 8 | 7-9 | 76 | 4 | 68 | 5 | 3-8 | 41 | 30 | 37 | No |
| 45. Knee pain when doing sports or exercise | 8 | 7-9 | 75 | 3 | 65 | 6 | 5-8 | 49 | 13 | 39 | No |
| 46. Recent knee pain intensity | 8 | 6-9 | 72 | 4 | 71 | 6 | 4-7 | 33 | 18 | 39 | No |
| 47. Average knee pain intensity | 8 | 6-9 | 71 | 0 | 70 | 8 | 7-9 | 79 | 5 | 39 | Yes |
| 48. Knee pain that occurs at night | 8 | 6-9 | 71 | 3 | 69 | 8 | 6-9 | 71 | 0 | 38 | Yes |
| 49. Knee pain when straightening the knee (extension) | 8 | 6-9 | 70 | 4 | 70 | 7 | 6-8 | 57 | 3 | 37 | No |
| 50. Knee pain intensity right now | 8 | 6-9 | 70 | 6 | 71 | 6 | 4-8 | 46 | 21 | 39 | No |
| 51. Knee pain that occurs later in the day | 8 | 6-9 | 70 | 0 | 70 | 6 | 5-7 | 37 | 5 | 38 | No |
| 52. Knee pain when changing position | 8 | 6-9 | 69 | 3 | 68 | 6 | 5-7 | 49 | 11 | 37 | No |
| 53. Knee pain when sitting | 7 | 6-9 | 65 | 3 | 69 | 7 | 6-8 | 74 | 5 | 39 | No |
| 54. Intermittent knee pain (pain that comes and goes) | 7 | 6-9 | 65 | 0 | 68 | 7 | 6-8 | 72 | 3 | 39 | No |
| 55. Knee pain when lying | 7 | 6-9 | 65 | 4 | 68 | 7 | 6-8 | 67 | 13 | 39 | No |
| 56. Whether knee pain interferes with sport | 8 | 6-9 | 63 | 3 | 67 | 6 | 5-8 | 40 | 11 | 38 | No |

^#^Number of participants rating each pain feature

*Interquartile range (25%-75% percentile)

**TableS3: Delphi study Round 2 (ordered by the percentage of the patient panel giving an importance rating of 7-9)**

|  | **Patient panel (n=67)** | | | | | **Clinician panel (n=35)** | | | | | **Retained in Round 3** |
| --- | --- | --- | --- | --- | --- | --- | --- | --- | --- | --- | --- |
|  | Median rating | IQR* | % 7-9 rating | % 1-3 rating | Number^#^ | Median rating | IQR* | % 7-9 rating | % 1-3 rating | Number^#^ |  |
| 1. Whether pain has improved since the operation | 9 | 8-9 | 95 | 2 | 64 | 9 | 8-9 | 97 | 0 | 35 | Yes |
| 2. Whether knee pain interferes with quality of life | 9 | 8-9 | 95 | 2 | 66 | 9 | 8-9 | 94 | 0 | 35 | Yes |
| 3. Whether knee pain is disabling | 9 | 8-9 | 95 | 0 | 66 | 8 | 7-9 | 80 | 0 | 35 | Yes |
| 4. Whether knee pain is controllable | 9 | 8-9 | 94 | 1 | 67 | 8 | 7-8 | 94 | 0 | 35 | Yes |
| 5. Whether knee pain is unbearable | 9 | 8-9 | 94 | 1 | 67 | 8 | 7-8 | 80 | 3 | 35 | Yes |
| 6. Knee pain when descending stairs | 9 | 8-9 | 94 | 0 | 65 | 8 | 7-8 | 80 | 0 | 35 | Yes |
| 7. Ability to cope with pain | 9 | 8-9 | 93 | 1 | 67 | 8 | 8-9 | 94 | 0 | 35 | Yes |
| 8. Whether knee pain interferes with walking | 9 | 8-9 | 92 | 0 | 66 | 8 | 8-9 | 100 | 0 | 35 | Yes |
| 9. Fear of moving knee because of the pain | 9 | 8-9 | 92 | 3 | 65 | 8 | 7-9 | 91 | 0 | 35 | Yes |
| 10. Worst knee pain intensity | 9 | 8-9 | 92 | 0 | 66 | 8 | 7-8 | 86 | 0 | 35 | Yes |
| 11. Ability to carry on despite the pain | 9 | 8-9 | 92 | 2 | 66 | 8 | 7-8 | 86 | 0 | 35 | Yes |
| 12. Knee pain when climbing stairs | 8 | 7-9 | 92 | 0 | 65 | 7 | 7-8 | 83 | 0 | 35 | Yes |
| 13. Knee pain when kneeling | 9 | 8-9 | 92 | 2 | 64 | 5 | 4-7 | 26 | 15 | 35 | Yes |
| 14. Frequency of pain medication use | 9 | 8-9 | 91 | 3 | 66 | 8 | 8-9 | 97 | 0 | 35 | Yes |
| 15. Dose of pain medication taken | 9 | 8-9 | 91 | 2 | 66 | 8 | 7-9 | 83 | 0 | 35 | Yes |
| 16. How long the pain has been present since the operation | 9 | 8-9 | 91 | 1 | 67 | 8 | 7-8 | 80 | 0 | 35 | Yes |
| 17. Whether knee pain interferes with rest or sleep | 9 | 8-9 | 89 | 2 | 65 | 9 | 8-9 | 97 | 0 | 35 | Yes |
| 18. Knee pain when walking | 8 | 7-9 | 89 | 0 | 66 | 8 | 8-8 | 97 | 0 | 34 | Yes |
| 19. Constant knee pain (pain that is there all the time) | 9 | 8-9 | 89 | 2 | 66 | 8 | 8-9 | 94 | 0 | 35 | Yes |
| 20. How pain compares to pre-operative pain | 9 | 8-9 | 89 | 0 | 65 | 9 | 8-9 | 91 | 0 | 35 | Yes |
| 21. Satisfaction with pain relief from the operation | 9 | 8-9 | 89 | 2 | 64 | 8 | 8-9 | 91 | 0 | 35 | Yes |
| 22. Type of pain medication taken | 9 | 8-9 | 89 | 2 | 66 | 8 | 8-9 | 91 | 0 | 35 | Yes |
| 23. Whether knee pain interferes with valued social, family or leisure activities | 8 | 8-9 | 89 | 2 | 65 | 8 | 7-9 | 89 | 0 | 35 | Yes |
| 24. Frequency of knee pain | 8 | 7-9 | 89 | 0 | 64 | 8 | 7-9 | 86 | 0 | 35 | Yes |
| 25. Whether knee pain interferes with work or housework | 9 | 7-9 | 88 | 2 | 65 | 8 | 8-9 | 94 | 0 | 35 | Yes |
| 26. Whether expectations of pain relief have been met | 8 | 8-9 | 86 | 0 | 65 | 8 | 7-9 | 94 | 0 | 35 | Yes |
| 27. Whether knee pain interferes with valued everyday activities | 8 | 7-9 | 86 | 2 | 66 | 8 | 8-9 | 91 | 0 | 35 | Yes |
| 28. Location of knee pain (e.g. kneecap, back of knee) | 9 | 8-9 | 86 | 0 | 65 | 8 | 7-9 | 91 | 0 | 35 | Yes |
| 29. Reduced need for pain medications | 8 | 8-9 | 86 | 3 | 65 | 8 | 7-8 | 82 | 0 | 34 | Yes |
| 30. Pain quality (how the pain feels e.g. sharp, aching, cramping) | 9 | 7-9 | 86 | 0 | 65 | 7 | 7-8 | 77 | 0 | 35 | Yes |
| 31. Knee pain with general activity | 8 | 7-9 | 86 | 0 | 64 | 7 | 7-8 | 77 | 6 | 35 | Yes |
| 32. Knee pain that occurs at night | 8 | 7-9 | 80 | 2 | 65 | 8 | 7-9 | 86 | 0 | 35 | Yes |
| 33. Average knee pain intensity | 8 | 7-9 | 76 | 0 | 67 | 8 | 7-8 | 80 | 0 | 35 | Yes |

^#^Number of participants rating each pain feature

*Interquartile range (25%-75% percentile)

**TableS4: Delphi study Round 3 (ordered by the percentage of the patient panel giving an importance rating of 7-9)**

|  | **Patient panel (n=62)** | | | | | **Clinician panel (n=33)** | | | | | **In core outcome set** |
| --- | --- | --- | --- | --- | --- | --- | --- | --- | --- | --- | --- |
|  | Median rating | IQR* | % 7-9 rating | % 1-3 rating | Number^#^ | Median rating | IQR* | % 7-9 rating | % 1-3 rating | Number^#^ |  |
| 1. Knee pain when walking | 9 | 8-9 | 95 | 0 | 61 | 8 | 8-9 | 97 | 3 | 33 | Yes |
| 2. Whether knee pain is unbearable | 9 | 8-9 | 95 | 0 | 60 | 8 | 6-9 | 67 | 9 | 33 | Yes |
| 3. Ability to cope with pain | 8 | 8-9 | 93 | 0 | 61 | 8 | 7-9 | 85 | 0 | 33 | Yes |
| 4. Ability to carry on despite the pain | 8 | 7-9 | 93 | 0 | 61 | 8 | 7-8 | 82 | 3 | 33 | Yes |
| 5. Whether knee pain interferes with walking | 9 | 8-9 | 92 | 2 | 62 | 8 | 8-9 | 97 | 3 | 33 | Yes |
| 6. Constant knee pain (pain that is there all the time) | 9 | 8-9 | 92 | 0 | 60 | 8 | 8-9 | 94 | 0 | 33 | Yes |
| 7. Knee pain when descending stairs | 9 | 8-9 | 92 | 0 | 59 | 8 | 7-8 | 85 | 3 | 33 | Yes |
| 8. Location of knee pain (e.g. kneecap, back of knee) | 9 | 8-9 | 92 | 0 | 61 | 9 | 7-9 | 82 | 0 | 33 | Yes |
| 9. Worst knee pain intensity | 9 | 8-9 | 92 | 2 | 61 | 8 | 7-9 | 79 | 0 | 33 | Yes |
| 10. Whether knee pain is disabling | 9 | 8-9 | 92 | 3 | 62 | 8 | 6-9 | 73 | 6 | 33 | Yes |
| 11. Knee pain when kneeling | 9 | 8-9 | 92 | 0 | 61 | 6 | 4-7 | 27 | 21 | 33 | Yes |
| 12. Whether knee pain interferes with quality of life | 9 | 8-9 | 90 | 2 | 62 | 8 | 8-9 | 94 | 0 | 33 | Yes |
| 13. Fear of moving knee because of the pain | 9 | 7-9 | 90 | 3 | 61 | 8 | 8-9 | 85 | 6 | 33 | Yes |
| 14. Knee pain with general activity | 8 | 8-9 | 90 | 0 | 60 | 8 | 7-9 | 76 | 3 | 33 | Yes |
| 15. Knee pain when climbing stairs | 9 | 8-9 | 90 | 0 | 60 | 8 | 7-8 | 76 | 3 | 33 | Yes |
| 16. Pain quality (how the pain feels e.g. sharp, aching, cramping) | 9 | 8-9 | 90 | 0 | 61 | 8 | 6-9 | 67 | 6 | 33 | Yes |
| 17. Whether knee pain interferes with rest or sleep | 9 | 8-9 | 89 | 2 | 61 | 9 | 8-9 | 94 | 3 | 33 | Yes |
| 18. Whether knee pain interferes with work or housework | 8 | 7-9 | 89 | 0 | 62 | 8 | 7-9 | 94 | 3 | 33 | Yes |
| 19. Frequency of pain medication use | 8 | 8-9 | 89 | 2 | 61 | 8 | 8-9 | 91 | 3 | 33 | Yes |
| 20. Whether pain has improved since the operation | 9 | 8-9 | 89 | 7 | 61 | 8 | 8-9 | 85 | 3 | 33 | Yes |
| 21. How pain compares to pre-operative pain | 8 | 7-9 | 89 | 3 | 61 | 8 | 8-9 | 82 | 3 | 33 | Yes |
| 22. How long the pain has been present since the operation | 9 | 7-9 | 89 | 0 | 61 | 8 | 7-9 | 82 | 3 | 33 | Yes |
| 23. Dose of pain medication taken | 9 | 8-9 | 88 | 0 | 59 | 8 | 7-9 | 82 | 3 | 33 | Yes |
| 24. Whether knee pain interferes with valued everyday activities | 8 | 7-9 | 87 | 0 | 62 | 8 | 7-9 | 91 | 3 | 33 | Yes |
| 25. Satisfaction with pain relief from the operation | 8 | 8-9 | 87 | 7 | 61 | 8 | 7-9 | 85 | 3 | 33 | Yes |
| 26. Whether knee pain is controllable | 8 | 7-9 | 87 | 2 | 61 | 8 | 7-9 | 85 | 3 | 33 | Yes |
| 27. Frequency of knee pain | 9 | 7-9 | 85 | 0 | 61 | 8 | 8-9 | 88 | 0 | 33 | Yes |
| 28. Type of pain medication taken | 8 | 7-9 | 83 | 0 | 60 | 8 | 7-9 | 88 | 0 | 33 | Yes |
| 29. Whether knee pain interferes with valued social, family or leisure activities | 8 | 7-9 | 83 | 0 | 60 | 8 | 7-9 | 85 | 3 | 33 | Yes |
| 30. Whether expectations of pain relief have been met | 8 | 7-9 | 82 | 5 | 61 | 8 | 7-9 | 76 | 0 | 33 | Yes |
| 31. Reduced need for pain medications | 8 | 7-9 | 80 | 3 | 61 | 8 | 7-9 | 76 | 6 | 33 | Yes |
| 32. Average knee pain intensity | 8 | 7-9 | 79 | 0 | 61 | 8 | 7-9 | 91 | 0 | 33 | Yes |
| 33. Knee pain that occurs at night | 8 | 7-9 | 78 | 3 | 60 | 8 | 7-9 | 85 | 3 | 33 | Yes |

^#^Number of participants rating each pain feature

*Interquartile range (25%-75% percentile)

**Table S5: Refinements to 8-item core outcome set after review by the Project Steering Committee (SC) and patient and public involvement group (PEP-R)**

| **Core outcome** | **Pain features within core outcome** | **Changes from preliminary core outcome** |
| --- | --- | --- |
| **Pain intensity** | Average pain intensity  Worst pain intensity  Whether pain is controllable | ‘Whether pain is controllable’: moved from emotional aspects of pain core outcome (PEP-R) |
| **Pain interference with daily living** | Whether pain interferes with work or housework  Whether pain interferes with valued everyday activities  Whether pain interferes with valued social, family or leisure activities  Whether pain interferes with walking  Whether pain interferes with quality of life  Whether pain interferes with rest or sleep | Core outcome name changed from ‘pain interference’ (SC) |
| **Pain and physical functioning** | Pain with general activity  Pain when walking  Pain when kneeling  Pain when climbing stairs  Pain when descending stairs  Whether pain is disabling | ‘Whether pain is disabling’: moved from pain interference with daily living core outcome (SC) |
| **Temporal aspects of pain (time and pain)** | Frequency of pain  Duration of pain since the operation  Night pain  Constant pain | Additional clarification of core outcome name added in brackets (SC) |
| **Pain description** | Pain location  Pain quality (e.g. sharp, aching, throbbing) | Core outcome name changed from ‘pain quality’ (PEP-R) |
| **Emotional aspects of pain** | Whether pain is unbearable  Pain self-efficacy  Ability to cope with pain  Kinesiophobia | Core outcome named changed from ‘pain-related emotional functioning’ (SC) |
| **Use of pain medications** | Frequency of pain medication use  Reduced need for pain medications  Type of pain medication taken  Dose of pain medication taken | No changes made |
| **Improvement and satisfaction with pain relief** | Whether expectations of pain relief have been met  How pain compares to pre-operative pain  Whether pain has improved since the operation  Satisfaction with pain relief from the operation | Core outcome name changed from ‘participant rating of improvement and satisfaction with pain relief’ (SC) |
